# Removal of the Outer Plate of the Skull Following a Severe Burn

**Published:** 1884-06

**Authors:** W. B. Woodall

**Affiliations:** Dentist


### Removal of the Outer Plate of the Skull Following a Severe Burn.

BY W. B. WOODALL, DENTIST.

Editor Dental Register:—In compliance with your request I will write for the Register the history of the operation I performed upon my uncle's head.

My uncle (T. P. Woodall) has been afflicted with epilepsy for eight years, and while sitting alone in his room, on the 22nd day of January, 1882, he was attacked with an epileptic fit, and fell, his head lying on the glowing coals in the fire place. He must have remained in this position ten minutes at least. He was first discovered by his wife, who was in the last stage of consumption, and though very weak, succeeded in dragging his head from the fire. I reached the scene in a few minutes. A young physician (Dr. F. B. Hunter) and I dressed the burn with linseed oil and lime water; we then gave him a heavy dose of morphine. Dr. S. L. Rountree, being my uncle's family physician, was called in as soon as possible, and he applied white lead to the entire burn. I must say I don't like to treat a burn with white lead, but give preference to the linseed oil and lime water. My uncle's hands were both burned, and his neck, face, and head were badly burned.

Dr. Rountree continued to treat the case for several weeks. No person thought he could possibly live twenty-four hours when he was first burned, and we expected him to die for months. His face was greatly swollen, and his eyes were closed for three weeks, so we could not tell whether his sight was destroyed or not. While in this condition, and for several weeks, Dr. Rountree kept him on a preparation of iron. In three or four weeks the burned flesh began to slough from his face and head. His left eye was destroyed, and the lids were entirely burned off. After the sloughing process was completed it left the frontal bone, the two parietal bones, a portion of each of the temporal bones, and a portion of the occipital bone exposed. We had no idea what the result would be, as history gave no account of such a case. We anxiously waited to see what nature would do. He suffered a great deal with his head and eyes. In eight or ten weeks from the time he was burned, we began to notice that necrosis was setting up all around the edge of the bones, where the exposed part of the bones connected with the portion that was covered with flesh. Exfoliation then commenced, and in a short time the entire bone (that portion that was exposed) was disconnected from the living bone. We then began to notice that there was a membrane of some kind forming under the edge of the bone.

Our hope, then was that the inner table was not hurt, and that nature would throw off the outer table and produce a membrane to shield the inner table; this was done by the aid of surgery to remove the outer table. I am satisfied that nature would have thrown off the dead bone, if his system could have borne up under the suppuration, but that was so great that the physicians decided that the bone would have to be removed, as fast as possible. No one seemed disposed to undertake the operation. Having never seen anything of the kind done, and not being over twenty-five years old, I felt a delicacy in undertaking the operation. My uncle was under my care, and I saw there was no hope of his living if the bone was not removed, so I concluded to undertake the operation. My first idea was to cut through it with the dental engine, but I was afraid I could not control it, and perhaps it might go too deep, so I decided to use something else. I finally concluded that the shape of a hand-saw file was such as to make it a safe instrument, with which to remove the bone; so I procured one and commenced the operation. This was in December, 1882, and it was completed in March, 1883. The first that I removed was the small portion of the occipital that was exposed. I next took a part of the frontal, and last the two parietal. I would file through and take off just as much as I thought it would be safe to take at one time. I would strike the bone with an instrument, and where the membrane had formed, the bone would sound as though it was hollow, and by this means I could tell how much it would be safe to attempt to remove. In fifteen months from the time he was burned I completed the operation. After which I used subnitrate of bismuth and vassaline on the sore to heal it. He has a complete membrane (or flesh) formed over the entire inner table. His head is not entirely healed yet, but is rapidly healing. His general health is good, and his mind is almost as clear as it ever was. His head and face are badly disfigured. The frontal bone, coming away, left the frontal sinus exposed, which leaves two large open deep pockets in his forehead. While his head was suppurating it was quite offensive; for this I used a weak solution of carbolic acid and listerine. He has had a few epiletic fits since he was burned, but not so hard nor as often as before.

---

The case here recorded was presented by Dr. Woodall at the recent meeting of the Alabama State Dental Society. It was examined with great interest; indeed, with astonishment. None present had ever seen or heard of anything approaching it in extent and severity.

The outer plate of the skull separated completely from the inner, taking one of the supercilliary ridges, and part of the other, and passing round the head, just below the superior edge of the rim of the ear, and passing round just below the occipital protuberance, where the lines of separation met. All above this line came away in six sections, bringing with it part of the diploe. These six sections are cemented together, and present the original shape of the head. Granulation took place from the border line of separation, grew and extended until it completely covered the portion from which the exfoliation had taken place, and scar tissue is being formed, which will doubtless ere long present the smooth cicatricial surface.

The extent and rapidity of granulation was truly surprising. And that such a catastrophe should happen to the head, without involving the brain and destroying life, seems almost miraculous. Physicians and surgeons decided that he must die, and refused to attempt any operation, regarding it as worse than useless. Dr. Woodall certainly is entitled to great credit for his treatment of the case; and especially so, when we consider that he is a dentist, and in the early period of his professional life; but he is more, he is a surgeon and a philosopher.

He has been advised to bring the case more directly to the attention of the medical profession; and for that purpose, no doubt, medical societies or colleges could make arrangements to have the case brought before them. It is certainly a very instructive case.—Ed. Register.

The above should have appeared in the May Register, but it was crowded out.

